# Health shocks and earnings trajectories: A comparative study of migrants and natives in Finland

**DOI:** 10.1016/j.jmh.2025.100387

**Published:** 2025-12-16

**Authors:** Waseem Haider, Laura Salonen, Elina Kilpi-Jakonen

**Affiliations:** a INVEST Research Flagship / Faculty of Social Sciences, University of Turku, Publicum 494 Assistentinkatu 7, 20014, Turku, Finland; b Finnish Institute of Occupational Health, Topeliuksenkatu 41, b00250, Helsinki, Finland; c INVEST Research Flagship / Faculty of Social Sciences, University of Turku, Publicum 472 Assistentinkatu 7, 20014, Turku, Finland

**Keywords:** Hospitalization, Earnings trajectories, Region of origin, Labor market outcomes, Difference-in-differences, Longitudinal analysis, Register data

## Abstract

**Objective:**

The earnings gap between migrants and natives is well-documented, but the extent to which health shocks contribute to this gap remains unclear. We estimated the impact of a health shock on long-term earnings for both natives and migrants from different regions of origin.

**Methods:**

Using high-quality full-population register data, we followed all residents aged 25 to 58 years in 2011, who were employed and did not experience a health shock between 2011 and 2012. A health shock was defined as the unanticipated hospitalization in 2013–14. Annual earnings were followed from 2011 to 2018. Using an event-study approach, we estimated the average treatment effects on the treated using dynamic difference-in-differences models stratified by gender and region of origin.

**Results:**

Health shocks led to substantial and persistent earnings losses. On average, the health shock reduced earnings levels by 7.4 % for natives and 13.5 % for migrants compared to their pre-shock earnings levels. Among migrants, significant declines were observed in earnings among those from European & Western, Russia and the former Soviet Union, as well as from ‘other’ region of origin. By gender, the earnings penalty due to health shocks was greater for migrant men (17.7 %), 1.5 times that of their female counterparts (8.6 %).

**Conclusions:**

The overall migrant–native difference of about six percentage points was small. Even if the average earnings penalty of a health shock is similar across groups, health shocks could still contribute to migrant–native disparities if migrants are more likely to experience such shocks initially.

## Highlights

• Analysis of the effect of health shock on earnings among natives and migrants.
• Use of rich register data, event study design and Difference-in-Differences models.
• Experience of a health shock negatively affects earnings of natives and migrants.
• The adverse effect was higher among migrants than among natives.
• Employment conditions explain the differences more than the migrant status.

## 1 Introduction

The existing literature indicates that migrants and natives frequently exhibit a consistent gap in labor market outcomes, including employment rates and earnings development (Sarvimäki, 2017, 2011; Vaalavuo and Rask, 2024). Empirical evidence shows that the migrant-native earnings differential is shaped by the combined influence of migration year and cohort (Borjas, 1985), country of origin (Adsera and Chiswick, 2007; Bratsberg et al., 2018), citizenship status (Constant and Zimmermann, 2008; Kahanec and Zaiceva, 2009), gender and ethnic identity (Adsera and Chiswick, 2007; Gorinas, 2014; OECD, 2023), disparities in human and social capital (Guzi and Mikula, 2022; Huber and Mikula, 2019), and enduring discrimination in the labour market (Becker, 1971). The economic effects of health shocks have also been studied in various contexts (e.g., across diverse countries, demographic groups, and health conditions), showing reductions in employment and earnings following illness or injury (Fouquet et al., 2024; García-Gómez et al., 2013; Lenhart, 2019; Vaalavuo, 2021), including prolonged spells of unemployment and increased reliance on social assistance (Dobkin et al., 2018). Yet, despite robust evidence in the general population, a significant knowledge gap remains about whether and how these effects differ systematically between migrants and natives. This study addresses that gap using full-population register data over nine years by comparing the earnings trajectories of migrants and natives before and after experiencing health shocks.

We expect a differential effect of the health shock on earnings between migrants and natives based on the following insights from the literature. The healthy immigrant effect (HIE) theory posits that migrants initially have an advantage over natives in health outcomes, driven by positive selective migration (Helgesson et al., 2019; Holz, 2022; Ichou and Wallace, 2019; Kennedy et al., 2015). Some studies counter the HIE theory, particularly in the European context (Iglesias et al., 2003; Solé-Auró and Crimmins, 2008). Other studies that consider the country of origin have found selective support for the HIE. For instance, in a Swedish register-based study using an objective measure of health, Helgesson et al. (2019) found the effect among Western migrants, but not among non-Western migrants. Kennedy et al. (2015), using cross-sectional survey data from the USA, Canada, UK, and Australia and a subjective measure of health, found evidence supporting the effect in migrants from developing countries, but not among those from developed countries. While the HIE suggests an initial advantage, it does not necessarily shield migrants from the long-term economic impacts of health deterioration. In fact, when migrants encounter health shocks, their position in the labor market may result in steeper and more persistent earnings declines compared to those of natives. For instance, migrants often find themselves in physically demanding jobs (Dunlavy and Rostila, 2013) and exposed to poorer psychosocial working conditions than natives (Sterud et al., 2018), and particularly non-Western migrants tend to work in low-skilled and lower-paid jobs than natives (Behtoui et al., 2020; Ilsøe, 2016; Slavnic and Urban, 2018). Moreover, migrants’ limited familiarity with healthcare systems and welfare provisions may amplify the economic impacts of health shocks, further exacerbating existing labor market disparities compared to natives (Spallek et al., 2011). Therefore, whether or not the HIE is present can affect initial conditions; however, it is the combination of health status and structural disadvantage that determines long-term economic vulnerability following a health shock.

Migrant women often face a compounded disadvantage in the labor market, earning considerably less than migrant men (Adsera and Chiswick, 2007), exhibiting higher rates of sickness absence (Bengtsson and Scott, 2006), and facing a higher risk of exiting the labor market due to health problems (Haider and Salonen, 2023). These existing disparities in the labor market might be amplified by a health shock, leading to steeper and more persistent adverse effects on labor earnings. Therefore, we also investigated the effect of health shocks on earnings from a gender perspective.

Another significant contribution of our study on the relationship between health and earnings is the use of an objective measure of health. Most studies examining the effect of health shock rely on self-reported health data, which can introduce measurement bias and often exclude underrepresented groups, especially migrants (Dobkin et al., 2018; Fouquet et al., 2024; García-Gómez et al., 2013; Lenhart, 2019). Unlike previous studies that rely on surveys and self-reported health data, we use comprehensive register data from 2011 to 2018, which provides clinically verified diagnoses. This approach offers a long-term view of developments before and after the health shock, allowing for a more accurate assessment of health-related changes in earnings among migrant and native populations.

Finland provides a particularly useful setting for studying how health shocks affect earnings among migrants and native-born individuals. Its universal welfare and healthcare systems ensure consistent institutional conditions for everyone, while high-quality register data allow for precise measurement of both health and labor-market histories. At the same time, Finland’s relatively recent yet rapidly diversifying immigration makes it an insightful case for understanding integration within a Nordic welfare regime: despite strong social protections, significant disparities in employment and health between migrants and natives remain (Sarvimäki, 2017; Tervola, 2020). This combination of universal institutions and emerging inequalities makes Finland an ideal context for examining labor-market resilience following health shocks.

Given the considerable socioeconomic implications of health shocks and the rising share of migrants in labor markets across high-income countries, understanding these differential effects is of both theoretical and policy relevance. In this study, we investigate how health shocks affect the earnings trajectories of migrants and natives differently, and whether the effect of a health shock on earnings differs by gender, using high-quality register data and an objective measure of health. We employ a difference-in-differences approach with an event study design. Our primary goal is to identify the groups most likely to face earnings penalties following health shocks. By examining how health shocks affect earnings across diverse backgrounds, we gain insight into the relationship between migrant status, health, and earnings and contribute to the discussion on health inequities. This detailed understanding is crucial for creating social protection systems that not only ease the immediate adverse financial effects of illness but also break the cycle between poor health and long-term income disadvantage, ultimately leading to a more equitable and resilient workforce.

### 1.1 Institutional setting

Finland has a universal healthcare system financed through general taxation. However, users must bear some relatively high out-of-pocket costs, such as consultation fees and prescribed drug purchases (Tervola et al., 2021). Finland's hospital care—covering medical, surgical, and psychiatric services—is largely provided by the publicly funded municipal system and is documented in the national Care Register for Health Care (Hilmo). It is important to note that Finland has an extensive occupational health care sector that covers all employees. Our study focuses solely on these hospitalizations, i.e., only inpatient care visits.

Even though patients in Finland pay user fees that are universally subsidized, the financial burden is relatively low. During the study period (2011–2018), statutory co-payments for inpatient stays were capped by an annual ceiling ranging from 633€ to 683€. Once the ceiling is reached, public-sector care is free for the remainder of the calendar year, and municipalities may waive fees for low-income patients even before the cap is reached. As a result, out-of-pocket expenses are unlikely to lead to significant socio-economic differences in post-hospital labor-market outcomes. This allowed us to focus on the earnings implications of the health shock itself, rather than on inequality related to medical costs.

## 2 Data and methods

### 2.1 Material

Individual-level data on the total population were obtained from the linked registers of Statistics Finland and the Finnish Institute of Health and Welfare (THL). Statistics Finland provides rich information on migration, sociodemographic characteristics, and earnings. The information on hospitalisation and diagnosis was obtained from THL’s Hilmo. Since data is routinely collected from administrative sources, the only sources of attrition were emigration and death.

#### 2.1.1 Study population

In our study, 'migrant' refers to individuals born abroad and currently residing in Finland who do not have Finnish-born parents. The study population consisted of migrants and natives aged 25 to 58 who resided in Finland in 2011 and were hospitalized either in 2013, 2014 or in 2019. We excluded individuals who were not employed at the end of two consecutive years from 2011 to 2012, who were hospitalized between 2011 and 2012, who died or emigrated during the follow-up, who were of Finnish origin but born outside of Finland, and those who were born to migrant parents in Finland. As we describe in more detail below, we measured the health shock of individuals in 2013, 2014, and 2019, followed by earnings development from 2011 to 2018.

The analysis excluded individuals who emigrated during the follow-up period to ensure internal validity, as labor market outcomes after emigration cannot be recorded in the Finnish population registers. Following the treatment years (2013–2014), 843 natives and 25,651 migrants emigrated. Among them, 16 natives (1.9 %) and 259 migrants (1.0 %) emigrated after experiencing a health shock. Of these 259 migrants, 117 (45 %) came from European & Western countries or Russia/the Former Soviet Union, groups typically characterized by stronger labor market attachment and more stable integration paths. These figures suggest that health shocks were not a major factor in emigration and that post-shock emigrants primarily belonged to relatively advantaged origin groups. While some selection due to emigration may exist, any bias in the estimated effects is likely minor. The results should therefore be viewed as reflecting labor market trajectories among individuals who stayed in Finland during the observation period.

To assess whether mortality could bias the estimated post-shock earnings effects, we tracked all individuals who experienced a health shock in 2013 or 2014 (*n* = 32,516) within the analytic sample of 62,866 and followed them until 2019 or death. A total of 407 (1.25 %) individuals who experienced health shock in 2013/2014 died during the period. The main diagnoses among those who died were external injuries (31 %), malignant neoplasms (19 %), and mental and behavioral disorders (17 %). Mortality was slightly higher among natives than migrants, and gender differences were minimal. It suggests that selective mortality was limited and unlikely to substantially bias the estimated treatment effects. However, as deaths were more common among individuals in weaker labor-market positions, the main findings may slightly underestimate the long-term economic consequences of health shocks.

#### 2.1.2 Measure of outcome

The outcome variable was annual earnings in euros, including wages and salaries from paid work and self-employment income. Earnings were adjusted to 2019 prices using the Household Index of Consumer Prices. We analyzed earnings in levels rather than logarithmic form because the outcome includes zero values, indicating complete non-employment for some individuals. As argued by Chen and Roth (2024), average treatment effects based on log or log-like transformations, often interpreted as percentage effects, are not well-defined when the outcome can be zero and should not be interpreted as approximate percentage changes, since their magnitude depends on the outcome’s original units. This issue is especially relevant when the treatment affects the extensive margin, such as transitions between employment and non-employment. Furthermore, as shown by Bellemare and Wichman (2020), while the inverse hyperbolic sine transformation is defined at zero, it does not produce scale-invariant percentage effects because the implied elasticities vary with the outcome level. We therefore retained the level specification, which provides directly interpretable estimates of absolute income changes in euros and facilitates the analysis of cumulative earnings losses over time within the dynamic difference-in-differences framework.

#### 2.1.3 Health shock

To measure health shock, we used information on inpatient care, including hospitalization and Classification of Diseases and Related Health Problems (ICD-10) diagnoses. Researchers have used the term ‘health shock’ to refer to hospitalization due to unanticipated health conditions (Böckerman et al., 2025; Dobkin et al., 2018; Garciía Gómez and López Nicolás, 2006; Lenhart, 2019; Riphahn, 1999; Vaalavuo, 2021; Zucchelli et al., 2010). In our study, we defined health shock as a set of sudden, severe, and largely unanticipated hospitalizations. Health shock was operationalized as a binary indicator denoting whether an individual experienced hospitalization for two or more days in 2013/14 or 2019 due to acute and severe cardiovascular events (I10-I15 hypertensive malignant emergencies, I21-I22 acute myocardial infarction, I50 heart failure, I60-I64 stroke), cancers (C00-C97 malignant neoplasms), injuries and accidents (S00-T98 injuries, poisoning, and specific other external causes), severe mental health conditions with acute presentations (F10-F19 substance-related disorders, F20-F29 schizophrenia, schizotypal and delusional disorders), and severe acute manifestations of chronic conditions (E10-E14 diabetic ketoacidosis and hyperosmolar coma, J44 COPD with acute exacerbation, N17-N19 acute renal failure, K25-K28 peptic ulcer with perforation or hemorrhage). The prevalence of health shocks is presented in Table 1.

**Table 1:** Distribution (%) of health shocks by migrant and gender status in 2013/2014.

Diagnosis	All		Men		Women	
	Native (*n* = 31,426)	Migrant (*n* = 1240)	Native (*n* = 14,146)	Migrant (*n* = 548)	Native (*n* = 17,280)	Migrant (*n* = 692)
Acute and sever cardiovascular events	11	8	16	13	7	4
Cancers	23	22	17	17	26	25
Injuries and accidents	12	13	16	17	10	10
Severe mental disorders	42	47	35	39	41	54
Acute chronic conditions	12	10	16	14	8	6

*Note:* Percentages refer to the proportion of individuals with each primary diagnosis group of among those experiencing a health shock, stratified by migrant status and gender.

#### 2.1.4 Sociodemographic variables

All the sociodemographic variables, except gender, were subject to change over time. Age was grouped into the categories 25–36, 37–47, and 48–58. Gender was defined as a binary variable consisting of male and female categories. The marital status variable was categorized into three groups: unmarried, married, and divorced/widowed. The family structure variable was divided into five categories: living alone without children, living with a partner without children, living with a partner with children, and the ‘other’ category. Occupational class was derived from a streamlined Erikson–Goldthorpe–Portocarero (EGP) scheme and collapsed into five groups: higher service class (EGP I), lower service class (EGP II), small business owners and farmers (EGP IV), skilled manual and non-manual working class occupations (EGP III, V, VI), and low-skilled workers (EGP VII). This condensation maintains the essential distinctions of the original EGP framework while ensuring sufficient sample sizes in each class. The region variable was classified into five categories: Uusimaa (Capital Region), South, West, East, and North.

#### 2.1.5 Migrant-specific variables

##### 2.1.5.1 Migrant status

We used a dummy variable indicating whether an individual is a migrant. For a more detailed definition of migrant status, see the section “study population.”

##### 2.1.5.2 Region of origin

We adopted a classification method for migrants consistent with previous studies conducted in Finland (Haider and Salonen, 2023; Jauhiainen and Raivonen, 2020). All European countries and Western countries, i.e., the United States of America, Canada, Australia, New Zealand, and the UK, fell under the “European & Western countries” category. Russia and the former Soviet Union republics were grouped as “Russia/Former Soviet Union”, except for Estonia, Latvia, and Lithuania (included in the “European & Western countries” category). All the countries on the Asian continent were grouped together as “Asian countries” except those where one-third of all migrants emigrated for humanitarian reasons. Countries from around the globe, where one-third of all migrants emigrated for humanitarian reasons (UNHCR, 2010), were grouped into the “refugee-origin” region of origin. Finally, all remaining countries, including most of Africa, were combined into the “other” category.

##### 2.1.5.3 Length of stay

We gathered information regarding the duration of stay for migrants from Statistics Finland’s migration register. We determined the length of stay in Finland by subtracting the entry date from the calendar year. We categorized the lengths of stay into four groups: below 7 years, 7–12 years, 13–18 years, and over 18 years.

### 2.2 Empirical approach

To address the challenges of unobserved heterogeneity and selection bias in estimating the causal impact of health shocks on earnings, we employed an identification method similar to that of Fadlon and Nielsen (2019). In this quasi-experimental framework, the control group encounters the event (treatment) after the follow-up. In traditional matching approaches, individuals are paired based on observable characteristics, but the matched controls may never experience the event of interest. While they may resemble treated individuals on measured covariates, they may differ in unobserved ways, such as disease susceptibility or labor market resilience, that are relevant to the outcome. For example, in studies of health shocks, matched controls might not share the same prognosis or underlying health risks as those who fall ill. In contrast, the event study design addresses this limitation by using as controls those individuals who experience the same event at a later point in time. This ensures that both groups are drawn from a population with similar risk profiles, thereby strengthening causal inference and reducing bias from unobserved heterogeneity. Our treatment group comprises individuals who faced a health shock in 2013/14 (T), whereas the control group consists of those who experienced a health shock in 2019 (*T* + 5). To improve comparability between the treated and control groups, we applied entropy balancing to construct analytical weights that equalize the distribution of key pre-treatment characteristics (Hainmueller, 2012). The balancing was performed on age, gender, marital status, occupational class, family structure, and region of residence, and additionally on length of stay for migrants. This reweighting procedure ensures that the control group matches the treated group on these observed covariates, thereby reducing bias from compositional differences before the health shock. This allowed us to capture the dynamics of earnings changes, such as anticipatory declines, delayed impacts, or recovery patterns, rather than assuming a constant average effect.

With this strategy, a causal interpretation is considered plausible if the trends in outcomes are parallel prior to treatment. We evaluated the parallel assumptions for each region of origin. We found that the earnings trends for both treatment and control groups were parallel before the health shock (see Table A.1). The health shock did not influence earnings before its occurrence. There was no contamination from dynamic treatment effects, as each participant experienced the event only once and not concurrently with others.

### 2.3 Statistical analysis

In line with recent literature evaluating the causal effects of health shocks (Böckerman et al., 2025; Fadlon and Nielsen, 2019; Vaalavuo, 2021), we estimated the causal impact of a health shock on subsequent earnings using a staggered difference-in-differences (DiD) framework that accounts for treatment occurring in multiple cohorts (2013 and 2014). The main analyses were conducted using the regression adjustment (RA) estimator implemented in the xthdidregress command in *Stata 18*. This estimator, developed by Wooldridge (2021), produces unbiased average treatment effects on the treated (ATTs) under treatment-effect heterogeneity by aggregating cohort-specific estimates. The RA approach models untreated potential outcomes as a function of observed covariates, individual fixed effects, and time fixed effects, and then contrasts observed outcomes with predicted counterfactuals.

We used the following equation to estimate the ATT.

$$\;\;Y_{it}=\;\alpha_{i}+\lambda_{year}+\;\sum_{r=-2}^{5}\beta_{k}D_{i,t+k}+X_{it\prime }\gamma +\;\varepsilon_{it}$$

Where $Y_{it}$ is the outcome for individual *i* in year *t*, $\alpha_{i}$ individual fixed effects capturing all time-invariant characteristics, $\lambda_{year}$ year fixed effects, $D_{i,t+k}$ a set of event-time dummies indicating the number of years *k* before or after the health shock (with *k*=−1 as the reference period), $\beta_{k}$ the dynamic treatment effects (ATTs) at each event time *k*, $X_{it}$ vector of time-varying controls, and $\varepsilon_{it}$ an idiosyncratic error.

For robustness checks, we re-estimated all models (presented in Table 3) using the conventional two-way fixed effects (TWFE) specification. While TWFE models are widely used in DiD applications, they may produce biased estimates when treatment effects are heterogeneous across cohorts or vary dynamically over time (De Chaisemartin et al., 2020; Sun and Abraham, 2021). Accordingly, TWFE estimates are reported primarily for comparison with prior literature, whereas interpretation focuses on the RA results, which are robust to staggered treatment timing and treatment-effect heterogeneity.

The resulting ATT estimates represent the average causal effect of experiencing a health shock on earnings, conditional on treatment status. We analyzed separate models for migrants and natives (Table 3), as well as separate models for each region of origin (Table 3). We then analyzed these models further, categorizing them by gender for each region of origin (Table 3). Our study uses full population register data rather than a sample; confidence intervals and p-values are provided to reflect model-based uncertainty rather than sampling uncertainty. In full-population data, statistical uncertainty stems not from random sampling, but from factors such as model specification, unobserved heterogeneity, and design-based variability. Therefore, confidence intervals should be viewed as indicators of the robustness and accuracy of the estimates, rather than definitive tests of probabilistic inference. This method aligns with current best practices in register-based econometric research, where measures of uncertainty are used to gauge the stability of estimates across different specifications (Abadie et al., 2020).

We conducted several sensitivity analyses. One analysis excluded individuals hospitalized for mental health conditions, as these conditions might significantly affect earnings before hospitalization. Another analysis focused specifically on the self-employed, since migrants from non-European backgrounds often work as self-employed. A third sensitivity analysis incorporated an interaction term between treatment status and occupational class within the individual fixed-effects model. In the fourth analysis, we used total earnings, including social benefits, as the outcome to assess the buffering effect of the welfare system. Additionally, we examined the extensive margins to determine how much the effect on earnings is driven by unemployment or retirement.

## 3 Results

### 3.1 Descriptive results

The sample consists of 62,866 individuals, of whom 4 % were migrants (Table 2). In 2013/14, 32,666 (52 %) individuals experienced a health shock. The migrant population was, on average, slightly younger, more frequently married or divorced, more often employed in manual labor or self-employed, and more likely to live in the capital area compared to natives. The largest group of migrants originated from European & Western countries (31 %), followed by Russia/former Soviet Union (24 %), and ‘other’ countries (24 %), and the smallest group came from refugee-origin countries (6 %).

**Table 2:** The distribution of sociodemographic and work-related factors, as well as migrant-specific characteristics, in 2013 (the sample was restricted to those employed for two years 2011–12) (*N* = 62,866).

	Migrants				Natives			
	Control		Treated		Control		Treated	
	N	*%*	N	*%*	N	*%*	N	*%*
Total	1090	100	1240	100	29,110	100	31,426	100
*Gender*								
Men	604	55	548	44	14,378	49	14,146	45
Women	486	45	692	56	14,732	51	17,280	55
*Age (years)*								
25–36	309	28	380	31	6784	23	8273	26
37–47	502	46	528	42	11,369	39	11,888	38
48–58	279	26	332	27	10,957	38	11,265	36
*Marital status*								
Unmarried	228	21	268	22	9477	32	10,622	34
Married	611	56	673	54	15,327	53	15,228	48
Divorced/Widowed	251	23	299	24	4306	15	5576	18
*Family structure*								
Alone, no children	116	15	213	17	5392	19	6957	22
Partner, no children	183	17	211	17	5832	20	6102	20
Partner, with children	507	47	517	42	14,184	49	13,436	43
Alone, with children	91	8	131	11	2177	7	2976	9
Other	143	13	168	13	1525	5	1955	6
*Occupational class*								
Higher service class	167	15	160	13	5862	15	5687	18
Lower service class	229	21	278	22	10,295	21	10,946	35
Small business owners and farmers	399	37	411	33	7041	37	7160	23
Skilled manual/non-manual workers	140	13	119	10	3528	13	3109	10
Low-skilled workers	155	14	272	22	2384	14	4524	14
*Region of residence*								
Capital region	579	53	667	54	9044	31	10,071	32
South	126	12	112	9	4370	15	3694	12
West	288	26	337	27	9621	33	11,458	36
East	61	7	64	5	2944	10	3194	10
North	36	3	60	5	3131	11	3009	10
*Region of origin*								
European & Western countries	342	31	408	33				
Russia/former Soviet Union	266	24	285	23				
Asian Countries	150	14	148	12				
Refugee-origin countries	74	7	78	6				
Other countries	258	24	321	26				
*Length of stay (years)*								
<7	199	20	185	15				
7–12	276	26	333	27				
13–18	220	18	245	20				
>18	395	36	477	38				

**Fig. 1:**
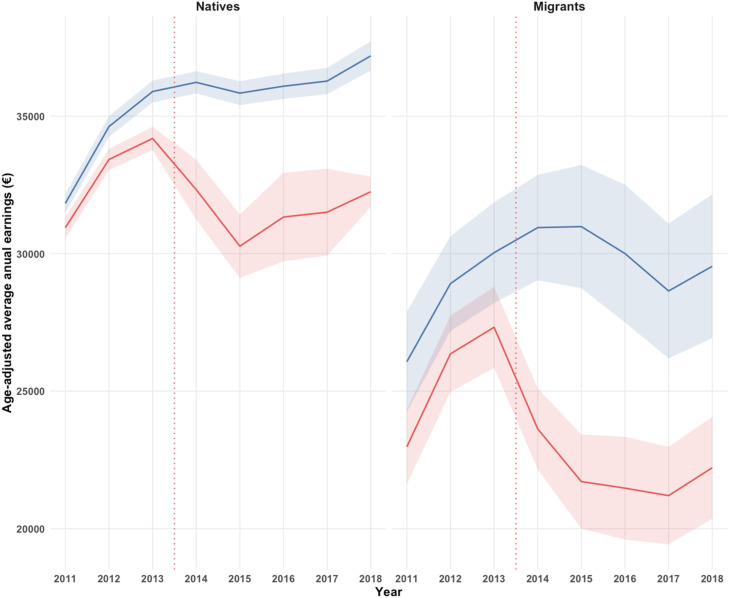
Age-adjusted average annual earnings of migrants and natives from 2011 to 2018 (with 95 % confidence intervals). The blue line represents earnings without a health shock, while the red line indicates earnings with a health shock. The dashed vertical line marks the health shock.

**Fig. 2:**
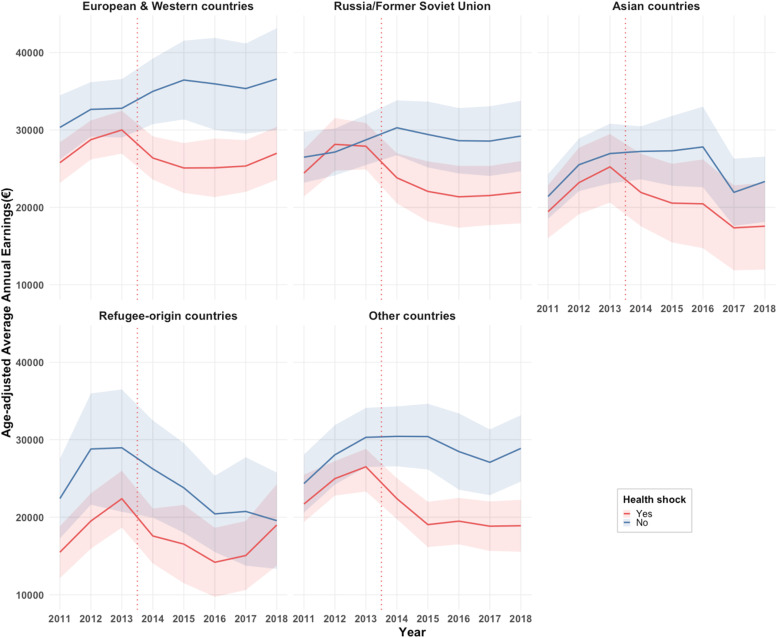
Average annual earnings in Euros by region of origin from 2011 to 2018 (with 95 % confidence intervals). Note: The vertical dashed line indicates a health shock.

Throughout the follow-up, the average annual earnings of natives were higher than those of migrants, regardless of health shocks (Fig. 1). Migrants from European & Western countries earned more on average than all other migrant groups regardless of a health shock (Fig. 2).

On average, earnings were consistently lower among those who had a health shock than those who did not, but the magnitude of the reduction varied by region of origin (Fig. 2). The lowest earnings were observed among migrants from refugee-origin countries and Asian countries following a health shock. The largest gap in earnings and earnings growth between those who experienced a health shock and those who did not was among migrants from ‘other’ countries.

### 3.2 The effect of health shock on earnings among migrants and natives

#### 3.2.1 Differences between migrants and natives

Both migrants and native workers experienced a significant reduction in annual earnings following a health shock: migrants lost approximately 3958€ (SE 1291€, *p* < 0.001) annually, while natives experienced a loss of about 2618€ (SE 280€, *p* < 0.001) (Table 3). The percentage decrease in earnings relative to observed average earnings due to health shock was 13.5 % among migrants and 7.4 % among natives.^1^

**Table 3:** Fully adjusted Difference-in-Differences panel regression models estimating the effect of health shocks on earnings among migrants and natives.

**ATT** Health shock vs no health shock	**RA**	**TWFE**	**Dynamic effects with RA estimator**								**Obs.**
			−2	−1	0	1	2	3	4	5	
Natives	−2618.3*** (280.0)	−2330.0*** (222.3)	187.9 (164.6)	160.6 (111.8)	−2042.9*** (255.9)	−3517.1*** (300.8)	−2908.6*** (419.7)	−2952.5*** (420.9)	−2847.0*** (240.8)	−2094.5*** (308.3)	319,648
Men	−2494.5*** (547.0)	−2858.0*** (397.5)	170.7 (284.0)	50.9 (190)	−2032.3*** (519.0)	−3676.4*** (590.7)	−2944.5*** (848.8)	−3215.2*** (843.4)	−3265.4*** (391.6)	−2665.4*** (501.8)	151,352
Women	–2238.9*** (208.0)	−1836.1*** (224.0)	202.6 (174.1)	229.1 (128.3)	−1962.9*** (149.8)	−3138.5*** (219.7)	−2526.1*** (247.6)	−2273.7*** (266.7)	−1936.6*** (288.4)	−947.8* (367.2)	168,296
Migrants	−3957.8*** (1223.0)	−4911.4* (2040.2)	776.0 (878.5)	193.6 (504.4)	−2501.7*** (603.9)	−4150.7*** (1002.1)	−4544.6*** (1430.4)	−4878.9** (1548.5)	−4297.8* (1813.2)	−2767.0 (2128.4)	12,522
Men	−5350.1*** (1290.9)	−4539.7* (1906.6)	−206.9 (912.9)	541.3 (659.1)	−3137.0*** (805.0)	−5334.0*** (1233.3)	−6077.5*** (1507.5)	−6137.5*** (1638.3)	−6122.5*** (1819.4)	−4175.0 (2651.3)	6224
Women	–2114.5* (1052.8)	−1949.4 (1269.3)	675.0 (856.3)	−407.3 (649.6)	−1638.2* (820.2)	−3003.0* (1213.5)	−2838.3* (1270.1)	−2863.2* (1184.4)	−1869.5 (1343.1)	−973.2 (1866.9)	6328
*Migrants (region of origin)*											
European & Western	−4113.8* (1930.0)	−5819.5 (3314.4)	1076.8 (1453.9)	509.2 (932.3)	−1927.0 (1193.3)	−4202.4* (1801.0)	−5860.7** (2223.3)	−5129.7* (2469.4)	−4083.3 (2794.9)	−2807.1 (3832.8)	4080
Russia/former Soviet Union	−3924.9** (1256.1)	−4766.0** (1558.7)	1600.5 (1083.3)	−1542.0 (836.2)	−2417.6* (1140.5)	−3634.9* (1519.9)	−4300.8** (1528.3)	−4734.2** (1705.4)	−4502.6* (1843.6)	−3995.5 (2681.9)	2888
Asian Countries	−2569.5 (2587.5)	374.3 (3675.2)	2274.2 (2531.0)	1563.8 (1524.8)	−3145.8* (1667.0)	−2686.5 (2392.7)	−3736.4 (3144.2)	−3873.2 (3450.5)	−2103.0 (3495.0)	2093.4 (4876.5)	1568
Refugee-origin countries	−3649.2 (3173.7)	−5359.7 (5359.7)	436.9 (2483.2)	−4450.4 2616.9	−5577.0* (2199.5)	−5965.8* (2903.5)	−4424.8 (4413.8)	−4057.2 (4210.5)	−839.1 (4112.7)	1746.0 (5768.0)	840
Other	–3904.2* (1614.3)	−3749.7* (1848.6)	31.6 (1207.9)	884.4 (907.1)	−3282.3** (1129.8)	−5221.9** (1750.1)	−2694.5 (2077.7)	−4159.5* (1952.1)	−4696.6* (2165.1)	−2741.7 (3223.4)	3176

ATT=Average Treatment Effect on Treated with robust standard errors in parenthesis. RA=Regression adjustment estimator. TWFE=Two-way fixed-effects estimator.

*p-value<0.05, ** p-value<0.01, ***p-value<0.001.

**Fig. 3:**
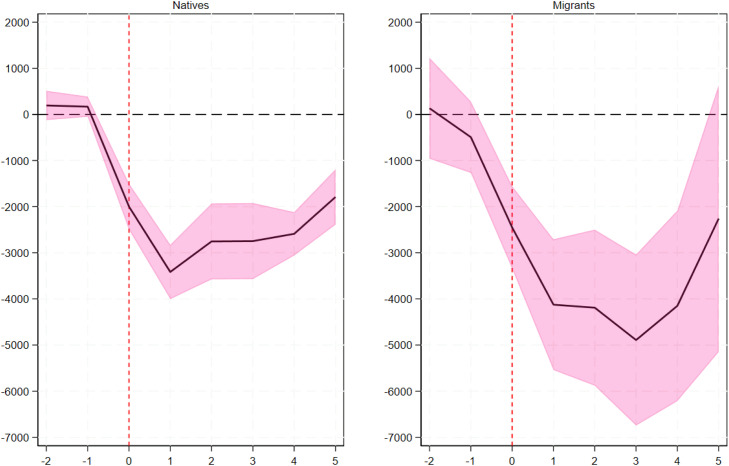
Fully-adjusted ATT of health shock with 95 % confidence intervals on average annual earnings of migrants and natives from 2011 to 2018. The dashed vertical line marks the health shock.

Among natives, health shocks consistently resulted in substantial and persistent negative effects on earnings, showing a pronounced decline for two years after a health shock with gradual but limited recovery over subsequent years (Fig. 3). In contrast, migrants experienced greater variability, initially facing substantial negative impacts, but demonstrated a noticeable, albeit partial, recovery from *t* + *+3* onward. The large confidence intervals for migrants indicate considerable statistical uncertainty and variability, highlighting heterogeneity in how health shocks impact migrant populations.

#### 3.2.2 Differences by region of origin

**Fig. 4:**
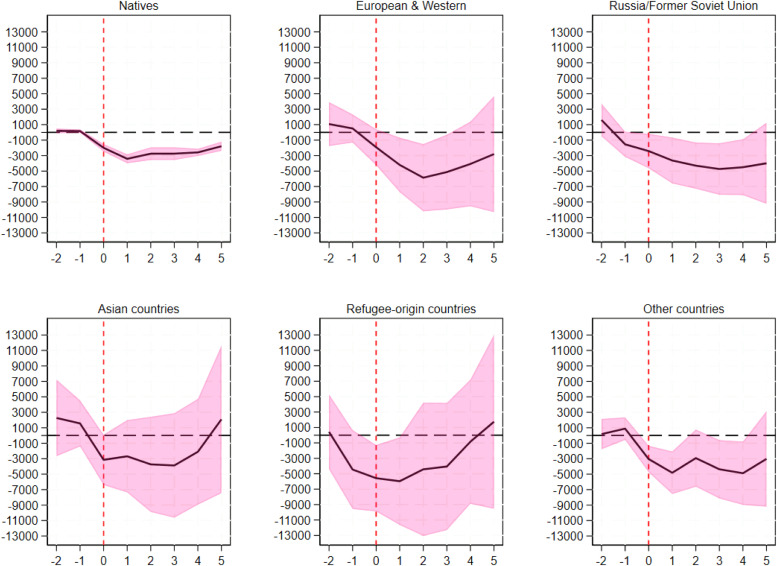
Fully-adjusted ATT of health shock on average annual earnings by region of origin with 95 % confidence intervals from 2011 to 2018. The dashed vertical line marks the health shock.

Among the six regions-of-origin groups, the annual earnings decline due to a health shock shows that the ATT was negative for all groups but reached a conventional significance level in migrants from European & Western countries, Russia/former Soviet Union, ‘other’ countries, and natives (Fig. 4). Migrants from European & Western countries experienced the steepest estimated loss of earnings after experiencing a health shock, the average loss in yearly earnings being approximately 4114€ (SE = 1930, *p* < 0.05), followed by migrants from Russia/former Soviet Union 3925€ (SE = 1256, *p* < 0.01) and migrants from ‘other’ countries 3904€ (SE = 1614, *p* < 0.05), while among natives the negative effect size was 2618€ (SE 280€, *p* < 0.001) (Table 3). The percentage decrease^2^ in earnings due to health shock relative to observed average earnings (without health shock) was 14 % among migrants from Russia/former Soviet Union and ‘other’ countries, and 12 % among migrants from European & Western countries. The variation in the effect of a shock on earnings among migrants indicates significant differences in labor market susceptibility to sudden health shocks across different migrant subpopulations.

We also observed substantial heterogeneity in both the magnitude and persistence of earnings losses after a health shock by regions of origin (Fig. 4). Migrant subgroups exhibited greater variability and statistical uncertainty. Migrants from European & Western countries as well as Russia/former Soviet Union experienced a sharper and more immediate earnings losses, which remained sustained over time. Migrants from Asian and refugee-origin countries experienced greater variability and wider confidence intervals, reflecting smaller sample sizes and more unstable employment trajectories. The larger post-shock dispersion likely reflects a mix of partial recovery for some and complete labor market exit for others, consistent with greater employment instability in these groups.

#### 3.2.3 Differences by gender

We also analyzed the effect of health shock by gender, but due to the small number of observations within regions of origin, we limited the analysis to ‘natives’ and ‘migrants’. Health shocks significantly reduced annual earnings for both native and migrant men and women, although the adverse effects were consistently greater for migrant men (Table 3). Among natives, men experienced an average decline of 6 % (–2494 €, SE=547, *p* < 0.001) in yearly earnings following a health shock, while the corresponding reduction for women was slightly larger at 7 % (–2239 €, SE=208, *p* < 0.001). Among migrants, however, the gender gap in vulnerability was far more pronounced. Migrant men experienced a 17.7 % (–5350 €, SE=1291, *p* < 0.001) decline in annual earnings, 1.5^2^ times the 8.6 % (–2114 €, SE=1053, *p* < 0.05) decline among migrant women.

The trajectory of ATT for earnings following health shocks differed by gender and migrant status. Among native men, earnings declined sharply immediately after the health shock, stabilized thereafter, and showed only partial recovery by the end of the five-year follow-up, indicating a persistent but moderate long-term earnings loss (Fig. A.1). The trajectory for migrant men revealed a markedly steeper and more prolonged income reduction. Earnings fell abruptly in the year following the health shock and continued to deteriorate over the next two years. Unlike natives, there is little evidence of post-shock recovery even five years after the event.

Among native women, following the health shock, yearly earnings declined sharply, reaching their lowest point two years after the event. Although a modest upward recovery occurred thereafter, earnings did not fully recover to pre-shock levels within the five-year follow-up period, suggesting lasting though moderate economic consequences (Fig. A.2). For migrant women, the decline in earnings was less precisely estimated but followed a broadly similar pattern. The post-shock reduction was smaller in magnitude, yet with greater variability in post-shock trajectories, likely reflecting heterogeneous labor market attachment and employment instability within this group.

### 3.3 Sensitivity analyses

Four sensitivity analyses were conducted to assess the robustness of the main findings. First, when health shocks due to mental disorders were excluded, the results remained largely unchanged (Table A.2). The estimated ATT for natives (–508€, SE=406) was small and statistically insignificant, whereas for migrants it remained sizeable (–2152€, SE=1046, *p* < 0.05), with the largest effect observed among migrant men (–3327€, SE=1046, *p* < 0.05). This suggests that the main earnings losses were not driven primarily by mental health events among migrants.

The second sensitivity analysis, restricted to self-employed individuals, showed considerably larger effects across groups. Among native men, the ATT was –3459€ (SE=892, *p* < 0.001), reflecting limited income protection in self-employment. Among migrants, estimates were more variable but indicated similar or greater vulnerability, particularly among those from Asian and Other origin countries. Overall, both analyses confirm that the adverse impact of health shocks on earnings is robust and particularly pronounced among self-employed men and migrants.

In the third sensitivity analysis, we included an interaction term between treatment status and occupational class in the individual fixed-effect model. Among natives, the size of earnings loss steadily grew smaller down the occupational hierarchy, starting with a small positive impact among upper service workers and leading to significant declines among small business owners, skilled manual workers, and especially low-skilled workers (see Table A.3). For migrants, notable decreases were also seen in low-skilled and manual jobs, though the losses were generally less severe.

In the fourth sensitivity analysis, we used the total earnings, including social benefits, as the outcome to estimate the buffering effect of the welfare statement system. When total earned income, including Social Security benefits, was used as the outcome, post-shock income losses were substantially smaller, particularly among natives (see Table A.4). Compared to the baseline model using only labor earnings, the estimated decline in total income decreased by about half for natives and roughly one-third for migrants. This reduction shows how Finland’s welfare system helps offset income loss by providing sickness, unemployment, and disability benefits. However, the level of protection varied among groups. While both native men and women experienced similar relative support, migrant men still faced nearly the same absolute loss as in the earnings-only model, indicating weaker access to or coverage by income-replacement programs.

The results of the extensive margins suggested that among natives, the likelihood of being employed dropped by 0.8 percentage points, and the likelihood of unemployment increased by a similar amount (both *p* < 0.001). Among migrants, the effects were in the same direction but not statistically significant (–1.6 percentage points for employment, +1.6 percentage points for unemployment, both *p* > 0.05). The impact on retirement could not be reliably measured due to very few transitions. These findings suggest that the overall earnings decline among migrants is not primarily due to leaving employment, but rather to lower earnings among those who remain employed.

## 4 Discussion

Our study examined how health shocks affect individual earnings within Finland’s universal welfare-state framework, comparing natives and migrants across gender. To our knowledge, no previous research has specifically examined how health shocks shape the long-term earnings trajectories of migrants using population-wide register data. While earlier studies, such as Vaalavuo (2021) and Fadlon and Nielsen (2019), have linked health and labor-market outcomes in the general population, evidence on how these dynamics differ between migrants and natives remains scarce. By focusing on migrants, our study contributes to understanding how health-related vulnerabilities reinforce earnings disparities and integration challenges within a comprehensive welfare-state context.

Our results show that health shocks substantially reduce annual earnings for all workers, but the magnitude of the loss varies across groups. Migrants experienced somewhat larger proportional declines than natives, although the difference was modest. This pattern suggests that Finland’s comprehensive welfare institutions buffer individuals against the full economic consequences of illness, consistent with evidence from other Nordic contexts (Helgesson et al., 2019; Vaalavuo, 2021). Yet, the buffering is incomplete. When total earned income, including taxable social benefits, was analyzed, estimated losses declined markedly for both groups, confirming that income-replacement schemes such as sickness, unemployment, and disability benefits partially compensate for lost earnings (Fadlon and Nielsen, 2019). However, the reduction in losses was smaller among migrants, especially migrant men, indicating that some groups benefit less from these protections, likely because they are over-represented in self-employment, temporary, or insecure contracts where benefit eligibility is weaker.

The interaction analysis between treatment status and occupational class further clarified these mechanisms. Among natives, the earnings penalty increased sharply down the occupational hierarchy, from small, statistically minor losses among upper-service workers to large, significant declines among low-skilled employees. Among migrants, a similar gradient emerged: manual and low-skilled workers experienced the largest penalties, while those in professional or administrative occupations were comparatively protected. These findings indicate that employment conditions, rather than migrant status alone, are central to understanding post-shock earnings vulnerability, echoing earlier research showing stronger relative impacts of health shock among workers in lower socioeconomic positions (Lenhart, 2019; Vaalavuo, 2021). Migrant disadvantage thus stems less from being foreign-born than from their concentration in poorer working environment and employment conditions. These employment structures heighten both health risks and the risk of income loss after a health shock.

From a gender perspective, these results challenge expectations based on previous research. Earlier studies have shown that migrant women are generally in weaker labor-market positions, face higher risks of sickness absence, and are more likely to leave employment after ill health (Adsera and Chiswick, 2007; Bengtsson and Scott, 2006; Haider and Salonen, 2023). Based on this, one might expect migrant women to experience greater post-shock earnings penalties. However, our findings show the opposite: migrant men faced larger proportional earnings declines after a health shock than migrant women, while the gender gap among natives was relatively small. This reversal suggests that men, on average, have limited access to flexible work options and job crafting compared to women. Migrant men’s stronger pre-shock attachment to the labor market may also intensify the perceived economic impact, as employment interruptions lead to sharper earnings drops. Overall, these patterns indicate that gender-based vulnerability among migrants is less about gender itself and more about the intersection of occupational exposure, job insecurity, and incomplete social insurance coverage.

Patterns across regions of origin reveal important differences in how health shocks affect economic outcomes. Migrants from European and Western countries and from Russia or the former Soviet Union experienced the most pronounced and persistent earnings declines, indicating that even relatively well-integrated groups are not immune to the long-term financial consequences of illness. In contrast, migrants from Asian and refugee-origin countries showed smaller and less statistically certain effects, which may reflect lower pre-shock earnings, greater reliance on social benefits, or household income pooling during periods of poor health. These findings suggest that the economic impact of ill health is influenced less by migrant origin itself than by underlying labor-market structures and employment conditions. Migrants with stronger labor-market attachment or higher-paying jobs tend to experience larger absolute losses because they have more to lose, whereas those in lower-paid or informal positions face smaller, yet still significant, declines that may be partly offset by welfare support. This pattern aligns with previous Nordic research showing that occupational position and job stability are more influential than migrant background in mediating the labor-market effects of health deterioration (Helgesson et al., 2019; Lenhart, 2019; Vaalavuo, 2021).

The results of our study offer a refined understanding of the HIE within the Finnish labor market. While the HIE suggests that migrants arrive with a health advantage due to positive selection (Helgesson et al., 2019; Ichou and Wallace, 2019; Kennedy et al., 2015), our findings indicate that this initial advantage does not shield them from the long-term economic repercussions of a health shock. In particular, migrant men experienced the largest proportional earnings losses following a health shock, despite likely benefitting from stronger pre-migration health selection and labor-market attachment. This pattern suggests that the protective effects of initial health advantage erode once migrants are exposed to the structural realities of the host labor market, such as employment in physically demanding or under precarious conditions, and constrained access to occupational health and rehabilitation services. In line with earlier Nordic evidence (Helgesson et al., 2019; Sterud et al., 2018), the results imply that the persistence of the HIE depends less on migrants’ baseline health and more on the social and institutional contexts that shape how illness translates into economic loss. Within Finland’s universal welfare framework, the diminishing returns of the HIE reflect not its absence but its conditionality, while good initial health may buffer early risks, unequal exposure to demanding work, lower-quality jobs, and informational barriers in navigating benefit systems ultimately determine the degree of resilience after a health shock.

## 5 Conclusion

Health shocks significantly decreased annual earnings for all workers, though the extent of the loss varied across different groups. Among native workers, earnings declines were similar for both genders, whereas, contrary to our expectations, among migrants, the decline was more pronounced for men. These patterns reveal two key insights. First, serious health events have substantial economic impacts on everyone, not just migrants. Second, the highest vulnerability appears among migrant men, likely because of their concentration in physically demanding and less secure jobs, while native men and women experience similar proportional declines. Although migrant men’s post-shock earnings losses exceeded those of natives, the estimated difference of about six percentage points was modest, and a formal test confirmed that the gap was not statistically significant.Even if the average earnings penalty of a health shock is similar across groups, health shocks could still contribute to migrant–native disparities if migrants are more likely to experience such shocks initially. This finding indicates that Finland’s universal welfare and income-protection systems help soften the financial impact of poor health across different groups. However, the apparent similarity between migrants and natives should not be mistaken for equal exposure or resilience. Migrants’ higher presence in lower-quality and physically demanding jobs likely increases their risk of income loss even within an inclusive welfare system. Taken together, these findings emphasize that labor-market vulnerability following health shocks is influenced more by employment conditions than by migrant status alone. Policies that improve job quality, support rehabilitation and workplace accommodations, and provide access to occupational health services, especially for those in manual and precarious jobs, could aid recovery and promote economic stability after illness. Ensuring equality in the Finnish labor market will depend on effectively extending these protections to all workers, regardless of their origin or gender.

## Strengths and limitations

The strengths of this study include high-quality full population register data, an objective measure of health, the exploration of the effects of health shocks by region of origin and gender, and the use of an event study approach and difference-in-differences analysis. The study's limitations include the fact that we could not include information on education in the study as it is not well covered in the registers for migrants, and we did not have information on migrants’ reason for arrival or admission residence permit category, which could affect their health and earnings (Pesola et al., 2024). We use broad categories of regions of origin because of the small number of migrants from specific countries. The different experiences of those migrants due to their ethnicity, culture, norms, and reasons for migration might be overshadowed by the dominant trends in a broad category. Our study does not directly address labor market discrimination against migrants, which could influence earnings. Lastly, due to the small number of observations, we were unable to conduct a detailed subgroup analysis by gender and region of origin.

## Ethics approval and consent to participate

This study was performed in line with the principles of the Declaration of Helsinki. This study was based on secondary data collected for administrative and statistical purposes. The study complies with the national legal framework for accessing pseudonymized personal data for scientific research carried out in the public interest. The informed consent was waived by the ethics committee of Statistics Finland Ethical (permission to access these data for this research purpose # TK/3279/07.03.00/2022); the legal basis is stated in the Finnish Personal Data Act (523/1999), Finnish Statistics Act (280/2004), and the EU General Data Protection Regulation (Art. 9 of the GDPR). Ethical approval was not required since the data were based on registers, according to Finnish Law.

## Data availability

Due to data protection laws and regulations, the data of this study are unavailable from the corresponding author. However, they are available from the register data holders (Statistics Finland and Finnish Institute of Health and Welfare) upon reasonable request and subject to fees.

## Funding

This research has been supported by the INVEST Research Flagship Center, funded by the 10.13039/501100002341Academy of Finland Flagship Programme [grant number:345546].

## CRediT authorship contribution statement

**Waseem Haider:** Writing – review & editing, Writing – original draft, Visualization, Software, Methodology, Investigation, Formal analysis, Data curation, Conceptualization. **Laura Salonen:** Writing – review & editing, Validation, Supervision, Methodology, Investigation, Conceptualization. **Elina Kilpi-Jakonen:** Writing – review & editing, Validation, Supervision, Project administration, Methodology, Investigation, Funding acquisition, Conceptualization.

## Declaration of competing interest

The authors declare that they have no known competing financial interests or personal relationships that could have appeared to influence the work reported in this paper.

## Appendix Supplementary materials

Image, application 1

## Footnotes

1 *Calculated as (ATT ÷ baseline average earnings without health shock) × 100*.

2 *Calculated as ((ATT_men – ATT_women) ÷ ATT_women) × 100*
