# Supplementary material for: Health shocks and earnings trajectories: A comparative study of migrants and natives in Finland
Source: J Migr Health. 2025 Dec 16;13:100387. doi: 10.1016/j.jmh.2025.100387 (PMC13404060; doi:10.1016/j.jmh.2025.100387)
Supplement: Supplementary file 1 [file mmc1.docx]

**Appendix**

**Table A.1.** Parallel-trends assumption tests (pretreatment time period) by region of origin

H_0_: Treatment effects in all the pretreatment periods are zero.

|  | **Chi2** | **Prob > chi2** |
| --- | --- | --- |
| Natives | 9.23 | 0.06 |
| European & Western | 1.92 | 0.59 |
| Russia/Former Soviet Union | 7.42 | 0.06 |
| Asian countries | 2.62 | 0.45 |
| Refugee-origin countries | 5.86 | 0.12 |
| Other countries | 4.04 | 0.26 |

Since the p-values were all above 0.05, we failed to reject the null hypothesis, indicating that the treatment effect was null in all pretreatment periods.

**Table A.2.** Fully adjusted Difference-in-Differences panel regression models estimate the effect of health shocks on earnings by migrant status and country of origin.

| **ATT**  Health shock vs no health shock | **Model specifications with RA estimator** | | | |
| --- | --- | --- | --- | --- |
|  | Excluding health shock due to mental health | Obs. | Self-employed individuals only | Obs. |
| Natives | –507.8  (405.7) | 231376 | -2409.6**  (702.2) | 39256 |
| Men | -671.4  (808.4) | 117056 | -3458.6***  (892.3) | 25416 |
| Women | -72.1  (233.0) | 114320 | -581.9  (1193.5) | 13840 |
| Migrants | -2152.1*  (1046.1) | 8680 | 639.9  (3839.2) | 1640 |
| Men | -3327*  (1445.4) | 4712 | 6990.8  (6181.1) | 952 |
| Women | -117.1  (1151.0) | 3968 | -3259.7  (8486.6) | 688 |
| European & Western countries | -1897.8  (2006.8) | 2736 | -6281.8  (6495.3) | 512 |
| Russia/Former Soviet Union | -1442.9  (1448.1) | 2120 | 3191.4  (6602.0) | 336 |
| Asian countries | -5041.9*  (2642.7) | 1144 | -654.9  (13043.3) | 296 |
| Refugee-origin countries | 3538.4  (5218.9) | 512 | 26153.3  (22158.2) | 80 |
| Other countries | -4242.3*  (1719.7) | 2168 | -1280.6  (7184.4) | 416 |
| _ATT=Average Treatment Effects on Treated with robust standard errors in parenthesis._  _RA= Regression adjustment estimator._  _*p-value<0.05, ** p-value<0.01, ***p-value<0.001_ | | | | |

**Table A.3.** Fixed-effects regression results showing differential earnings effects of health shocks on earnings by occupational class and migrant status.

|  | Natives | Migrants |
| --- | --- | --- |
| Treat X Occupational class |  |  |
| Upper services | 2987.1*  (1151.9) | 1719.1  (1654.9) |
| Lower services | -2923.9***  (302.7) | -160.2  (1582.0) |
| Small business owners and farmers | -7086.5***  (548.4) | -981.6  (2205.9) |
| Skilled manual/non-manual workers | -5288.5***  (308.5) | -1648*  (389.2) |
| Low-skilled workers | -18567.4***  (341.1) | -13876.2***  (1416.3) |

**Table A.4**. Average Treatment Effects on Treated (ATT) of health shock on earned income and total earned income (including social benefits), by migrant status and gender

|  | ATT (Earned income) | ATT (Total earned income, incl. benefits? | Change in effect |
| --- | --- | --- | --- |
| Natives | -2618.3***  (280.0) | -1121.3***  (299.8) | 53% smaller |
| Men | -2494.5***  (547.0) | -1270.1*  (607.9) | 49% smaller |
| Women | –2238.9***  (208.0) | –1145.4***  (171.2) | 49% smaller |
| Migrants | -3957.8***  (1223.0) | -2590.3*  (1158.8) | 35% smaller |
| Men | -5350.1***  (1290.9) | -5270.0*  (2389.9) | no reduction |
| Women | –2,114.5*  (1052.8) | –1218.4*  (848.8) | 42% smaller |


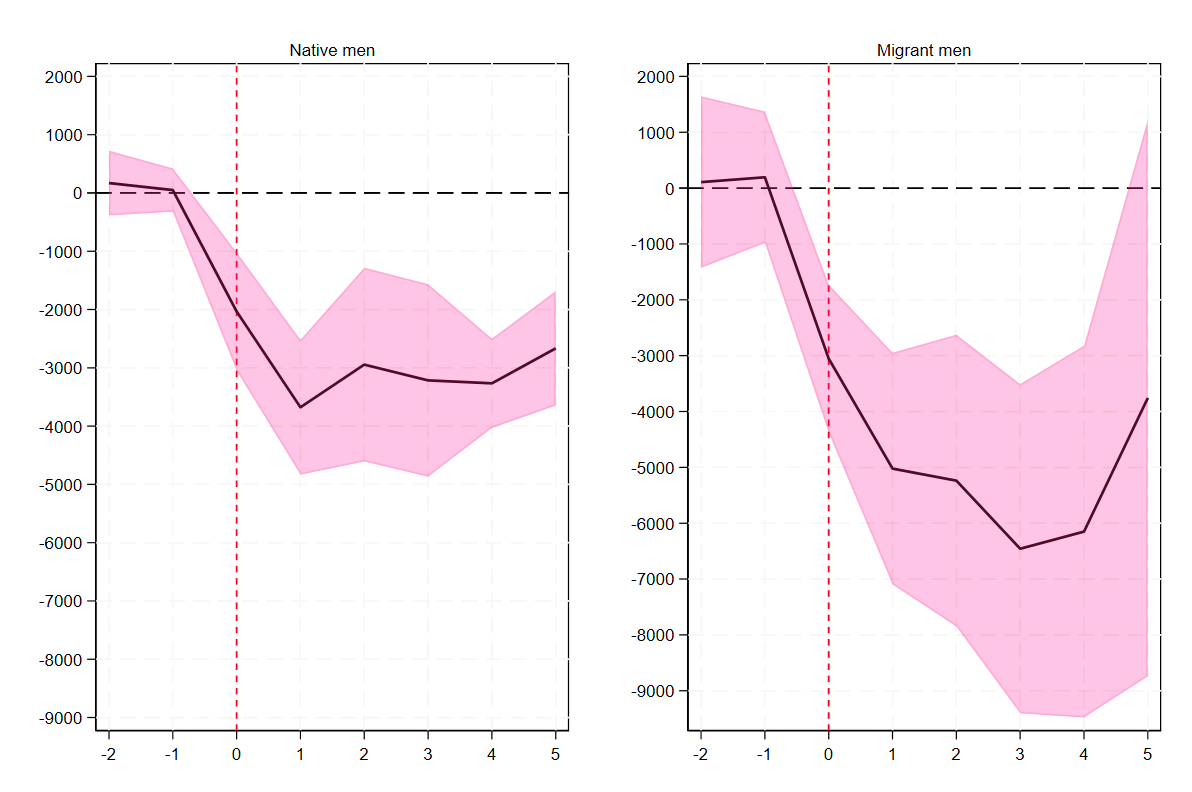
**Fig. A.1**. Fully-adjusted ATT of health shock with 95% confidence intervals on average annual earnings of migrant and native men from 2011 to 2018. The dashed vertical line marks the health shock.


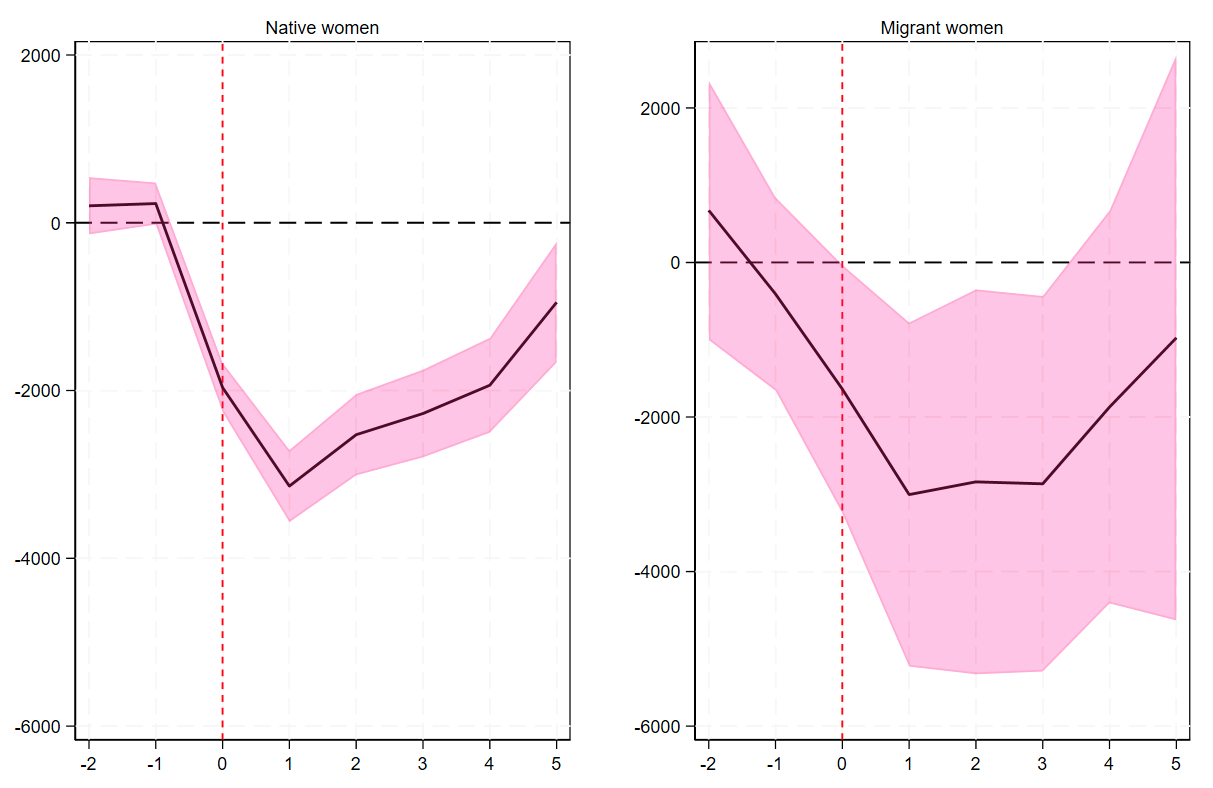


**Fig. A.2**. Fully-adjusted ATT of health shock with 95% confidence intervals on average annual earnings of migrant and native women from 2011 to 2018. The dashed vertical line marks the health shock.
